# Pharmacological Interactions Between Nutritional Supplements and Prescription Medications in Older Adults: A Comprehensive Review

**DOI:** 10.7759/cureus.92363

**Published:** 2025-09-15

**Authors:** Gichin Changaramkumarath, Jane M Abucha, Mekdes M Wollel, Nethra Somannagari, Alousious Kasagga, Aayam Sapkota, Iana A Malasevskaia

**Affiliations:** 1 Internal Medicine, Chongqing Medical University, Chongqing, CHN; 2 Medicine, American University of Integrative Sciences, Kennesaw, USA; 3 Pediatrics and Child Health, University of Gondar, Gondar, ETH; 4 Neurology, Gandhi Medical College, Hyderabad, IND; 5 Pathology, Peking University, Beijing, CHN; 6 Pathology, California School of Podiatric Medicine, Oakland, USA; 7 Hospital-Based Medicine, Harvard T.H. Chan School of Public Health, Boston, USA

**Keywords:** adverse drug events, diet-drug interaction, geriatric care, herbal supplements, medication safety, older adults, patient-provider communication, pharmacodynamics, polypharmacy, supplement-drug interactions

## Abstract

The global aging population faces increasing risks of supplement-drug interactions due to rising polypharmacy and widespread use of nutritional supplements. Older adults, particularly those with chronic conditions, frequently combine prescription medications with dietary supplements, yet healthcare providers often overlook these interactions, leading to preventable adverse effects. This review synthesizes evidence from 16 international studies spanning nearly three decades, examining the intersection of supplement and medication use in older adults. Key findings reveal a high prevalence of concurrent use (23-82.5%), significantly increasing the likelihood of adverse interactions, particularly with antithrombotics (e.g., warfarin and ginkgo) and absorption-disrupting minerals (e.g., calcium and levothyroxine). A critical systemic failure in patient-provider communication exacerbates these risks, as clinicians often neglect to inquire about supplement use. Despite widespread potential interactions, actual clinical harm appears concentrated in high-risk combinations. The review calls for proactive clinical strategies, including standardized supplement screening, targeted patient education, and pharmacist-led medication management. Its limitations include cross-sectional study designs and self-reported data, underscoring the need for longitudinal and intervention-based research. Future studies should prioritize causal evidence, standardized methodologies, and data from low- and middle-income countries to mitigate risks in aging populations.

## Introduction and background

The world’s population is aging at an unprecedented pace. By 2050, 22% of the global population will be over 60 years old, which is nearly double the proportion in 2015, and 80% of these older adults will reside in low- and middle-income countries [1]. This demographic shift is driven by declining birth rates and increased life expectancy, which reached 73.3 years globally in 2024. Individuals aged ≥65 years currently comprise 830 million globally, a population projected to double to 1.7 billion by 2054 [1]. As this population grows, so does the prevalence of age-related health concerns, chronic disease burden, malnutrition (often secondary to impaired nutrient absorption), and polypharmacy, collectively exacerbating risks for adverse drug reactions and food-drug interactions [2]. Nearly 91% older adults take at least one medication, and 81% use prescription drugs [3]. Over half take five or more medications, including prescriptions, over-the-counter drugs, or supplements, reflecting widespread polypharmacy. Additionally, about one in 25 people face a risk of a major drug interaction, with nonprescription medications contributing to half of these cases [3,4]. Concurrently, the use of complementary and alternative medicine (CAM), particularly nutritional supplements, has surged globally. This trend is especially pronounced among older adults, a demographic in which a striking 85% manage at least one chronic illness and 60% navigate the complex challenges of multiple comorbidities. However, despite its growing prevalence, CAM-related drug interactions remain poorly understood, and many healthcare providers remain hesitant to recommend CAM due to a lack of robust evidence [5]. A Veterans Affairs medical center study found that approximately 25% of older patients used dietary supplements, and more than 50% of these individuals faced potential interactions with their prescription medications. These findings underscore the importance of healthcare providers routinely reviewing patients' supplement use to mitigate medication risks [6]. A comparative study by Ben-Arye et al. found notable differences in how physicians oversee dietary and herbal supplement use [7]. Primary care physicians (PCPs) demonstrated greater awareness and engaged patients in discussions more frequently, whereas hospital physicians (HPs) were less informed and rarely initiated such conversations. These disparities underscore the critical need for enhanced care coordination to standardize supplement monitoring across healthcare settings, thereby minimizing preventable medication-related risks. [7]. This growing public health concern demands an evidence-based response. To meet this need, we conducted a literature review aimed to examine supplement-drug interactions in older adults with three key objectives: (1) identifying the most prevalent and clinically significant interactions, (2) elucidating their pharmacokinetic and pharmacodynamic mechanisms, and (3) evaluating both the clinical consequences and current gaps in research and clinical practice. Ultimately, this review seeks to provide evidence-based recommendations to optimize medication safety and guide clinical decision-making for healthcare providers managing geriatric patients.

## Review

A structured literature search was performed between May 12, 2025, and May 16, 2025, across three electronic databases: PubMed/MEDLINE, Europe PMC, and ScienceDirect. The search strategy was designed to capture studies relevant to nutritional supplements, older adult populations (defined as individuals aged 65 years and older), and drug interactions. Keywords and Medical Subject Headings (MeSH terms, where applicable) were combined using Boolean operators. Core concepts included "nutritional supplementation" (e.g., "dietary supplements," "nutraceuticals"), "aged adults" (e.g., "elderly," "geriatric population"), and "drug interactions" (e.g., "pharmacological interactions," "drug-supplement interactions," "herb-drug interactions"). Specific filters for language (English), human studies, and article type (e.g., full text, research articles, review articles, practice guidelines) were applied within each database interface to refine the search results. The detailed search strategy and filters applied for each database are presented in Table 1.

**Table 1:** Search strategy

Database/app	Search strategy	Filters used	Number of studies	Date
PubMed/MEDLINE	("Nutritional supplementation"[MeSH Terms] OR "Nutritional supplementation"[Title/Abstract] OR "Dietary supplements"[MeSH Terms] OR "Dietary supplements"[Title/Abstract] OR "Nutraceuticals"[MeSH Terms] OR "Nutraceuticals"[Title/Abstract]) AND ("Aged"[MeSH Terms] OR "Aged"[Title/Abstract] OR "Aged adults"[Title/Abstract] OR "Elderly"[Title/Abstract] OR "Geriatric population"[Title/Abstract] OR "Geriatrics"[MeSH Terms]) AND ("Drug interactions"[MeSH Terms] OR "Drug interactions"[Title/Abstract] OR "Pharmacological interactions"[Title/Abstract] OR "Drug-supplement interactions"[Title/Abstract] OR "Herb-Drug Interactions"[MeSH Terms])	Full text English, humans	172	5/15/2025
Europe PMC	ABSTRACT:((("nutritional supplementation" OR "dietary supplements" OR nutraceuticals) AND ("aged adults" OR elderly OR "geriatric population" OR aged OR geriatrics) AND ("drug interactions" OR "pharmacological interactions" OR "drug-supplement interactions" OR "herb-drug interactions")) )	Advanced search	111	5/15/2025
Science Direct	TITLE-ABS-KEY(("older adults" OR elderly) AND ("nutritional supplements" OR vitamins) AND ("drug interactions" OR pharmacokinetics))	Article type: research articles, review articles, practice guidelines	47	5/15/2025

All records identified from the database searches (n=330) were imported into the Rayyan app. [8], for duplicate removal and screening. After the removal of 24 duplicates, the titles and abstracts of the remaining 306 records were screened against predefined inclusion criteria. These criteria focused on studies reporting on the geriatric population using nutritional supplements and experiencing pharmacological interactions when combined with prescription medications. Studies were excluded if they did not meet these core parameters, were not in English, or were not primary research or relevant review articles/guidelines. Following title and abstract screening, 277 records were excluded, leaving 29 articles for further consideration or full-text review. After detailed evaluation, 16 studies met the inclusion criteria and were included in the final qualitative synthesis.

Summary of included studies

The included studies comprise a mix of original research and review articles. The original research was predominantly observational, including cross-sectional surveys, secondary analyses of trial data, and clinical audits (Table 2). These studies spanned multiple geographic regions, including the United States, the United Kingdom, Europe, and Asia, with publication dates ranging from 1997 to 2024. The original research was predominantly cross-sectional, with sample sizes ranging from 124 to over 15,000 participants. The findings from these 16 primary and secondary sources converge on three critical themes regarding the use of nutritional supplements in older adults: the high prevalence of use and polypharmacy, the identification of common supplement-drug interactions and high-risk patient groups, and the critical role of communication gaps in exacerbating these risks.

**Table 2:** Summary of included studies on supplement-drug interactions in older adults AMD: age-related macular degeneration; ASPREE: The ASPirin in Reducing Events in the Elderly trial; BPH: benign prostatic hyperplasia; CAM(s): complementary and alternative medicine(s); DRPs: drug-related problems; FS(s): food supplement(s); GP: general practitioner; INR: international normalized ratio; LMQ-3: Living With Medicines Questionnaire version 3; NSAIDs: nonsteroidal anti-inflammatory drugs; OTC: over-the-counter; PCNE: Pharmaceutical Care Network Europe; RDA: recommended daily allowance; Rx: prescription

Paper	Study details	Key findings: medication use, interactions, and high-risk groups	Clinical implications	Study limitations and bias
Aykan and Aykan (2018) [9]	Cross-sectional study of 343 patients with cardiovascular diseases in Turkey (July to December 2017). Data collected via a self-administered questionnaire	Medication Use: 82.5% of patients with chronic cardiovascular diseases use dietary herbal supplements. - Interactions: Garlic affects platelet function and blood coagulation; onions decrease glucose levels; walnuts improve endothelial function. - High-Risk Groups: Patients with hypertension, dysrhythmia, and smokers are more likely to use herbal supplements, increasing the risk of interactions with prescription drugs	Understanding interactions between herbal supplements and drugs is necessary to minimize adverse reactions. - Concomitant use of herbal supplements and prescription drugs can lead to adverse reactions. - Garlic and onion can interact with antihypertensive drugs, affecting blood pressure and coagulation. - Garlic affects platelet function and blood coagulation, requiring cautious use with anticoagulants. - Garlic has antidiabetic activity, and onion lowers glucose levels, requiring careful management in diabetes. - Physicians should inquire about herbal product use to identify potential interactions and educate patients about benefits and risks	Conducted at a single tertiary facility. - Potential under-reporting due to self-administered questionnaires. - Need for further studies on early consumption and toxicity management. - Need for large-scale clinical trials to investigate herb-drug interactions
Chan et al. (2012) [10]	Observational study of 193 older adults in Taiwan. Data collected through review of baseline data from Medication Safety Review Clinic patients, including prescription drug information and comprehensive geriatric assessments	High prevalence of DRPs: 87% of participants had at least one DRP. - High-risk groups: Older adults, those with orthostatic hypotension, and those taking more chronic medications. - Common DRP categories: "Drug not taken/administered" (35%), "Potential interactions" (12%), and "Inappropriate duplication of drug" (11%). - Most common offending medication categories: Cardiovascular agents (33%), psychotropics (24%), and alimentary tract agents (16%). - Polypharmacy is a significant risk factor for DRPs	High prevalence of DRPs among geriatric outpatients with polypharmacy necessitates careful medication review to improve prescription quality. - Collaboration between geriatricians and pharmacists is recommended for thorough evaluation and intervention. - Expansion of clinical pharmacist services from inpatient to outpatient settings is suggested. - Future studies are needed to assess the effectiveness of medication review in resolving DRPs and improving outcomes	Small sample size. - The high educational level of participants is not representative of the broader Taiwanese population. The PCNE classification system was newly introduced to Taiwan, potentially limiting comparability with other studies. - Underestimation of DRP prevalence due to focus on chronic medications only. - Need for future studies with population-representative samples
Ly et al. (2002) [6]	Survey of 124 patients at a Veterans Affairs medical center in the United States (2000-2001). Data was collected via surveys completed in the clinic, by mail, and by telephone	Prevalence of dietary supplement use: 23% of patients were current users. - Mean number of prescription medications: 6 ± 3. - Mean number of dietary supplements: 3 ± 2. - Most commonly used supplements: Garlic and glucosamine. - High-risk interactions: Ginkgo with aspirin, garlic with warfarin. - High-risk groups: Elderly patients taking multiple medications and supplements	Pharmacists must be aware of and warn elderly patients about potential interactions between dietary supplements and prescription drugs. - Providers should screen patients for potential interactions between drugs and dietary supplements. - Use of patient education pamphlets and computerized screening programs can help minimize interactions	There is a lack of strong literature support for many drug-dietary supplement interactions. - Small sample size and low response rate. - Potential bias or inaccuracy in self-reported data. - Limited applicability to the general population due to the convenience sample of predominantly male veterans
Zarowitz (2010) [11]	Review article	Saw palmetto can interact with BPH medications and should be discontinued before surgery due to platelet aggregation effects. - High doses of glucosamine and chondroitin can increase INR in patients taking warfarin. - Glucosamine and fish oil should be discontinued before surgery to avoid bleeding risks. - Melatonin requires careful use due to potential adverse effects and should be used at the lowest dose for the shortest time. - Caution is recommended due to inconsistent clinical evidence and uncertainty about supplement constituents	Saw palmetto: Avoid drug duplication with conventional BPH treatments, discontinue before surgery due to bleeding risk. - Glucosamine sulfate: Comparable to conventional analgesics, high doses may increase INR in warfarin users; discontinue before surgery. - Fish oil: Reduces cardiovascular risk; discontinue before surgery due to bleeding risk. - Lutein and zinc: May improve vision and reduce AMD progression, but lutein does not prevent AMD. - Melatonin: Effective for circadian rhythm and sleep, use with caution due to sedative effects. - Probiotics: Useful for gastrointestinal conditions, not interchangeable, caution advised due to inconsistent evidence. - General caution: Inconsistent clinical evidence and uncertainty about the constituents of dietary supplements	The information provided is not comprehensive. - Variability in effectiveness due to inconsistent content of commercially available products. - Lack of data supporting the combination of saw palmetto with conventional BPH treatments. - Conflicting findings and safety concerns regarding probiotics in adults. - Inconsistent clinical evidence and uncertainty about the constituents of dietary supplements
Canter et al. (2004) [12]	Self-completed questionnaire survey, UK, sample size of 271. Data collected via self-completed questionnaires requested by telephone, post, or email after advertisement on websites and in a magazine	Medication Use: The average number of herbal and nutritional supplements used was 5.51 for men and 6.08 for women. The average number of prescription drugs used was 2.36 for men and 2.25 for women. - Interactions: The 12 most frequently reported herbs have potential for negative interactions with commonly used prescription drugs. - High-Risk Groups: Older people are at risk due to frequent use of multiple herbal and nutritional supplements alongside prescription drugs, with poor reporting to doctors	The decision to use herbal medicine should be based on a careful risk/benefit analysis. - Herbal medicines pose risks of intrinsic toxicity, adulteration, and negative interactions with drugs or other herbs. - Older adults are at increased risk due to polypharmacy and potential interactions with prescription drugs. - Commonly used herbs have potential interactions with frequently prescribed drugs. - Poor reporting of herbal use to doctors increases the risk of negative interactions	Provides no data on the prevalence of use among the broader population. A sample drawn from a specific magazine's readership may not be representative. - Selection bias due to self-selection of participants. - Lack of demographic data beyond age and gender. - Subject to recall bias. - Small sample size may limit generalizability - Did not collect detailed information on timing or concomitant use of products
Sadowska et al. (2016 [13]	Cross-sectional survey of 146 women over 50 years of age in Poland. Data collected via questionnaires distributed in spring 2015	Medication Use: 88.4% of respondents took prescription drugs; 44.5% took OTC drugs; 66.4% took dietary supplements. - Interactions: 35.8% of women at risk for drug-supplement interactions; common combinations include calcium/iron with ß-blockers and levothyroxine. - High-Risk Groups: Women over 50 taking prescription drugs and dietary supplements concurrently; those not waiting at least two hours between taking drugs and supplements	Concurrent use of dietary supplements and drugs can lead to complications in pharmacotherapy due to interactions. - A high percentage of women over 50 take both prescription drugs and dietary supplements, increasing interaction risk. - Concurrent use without adequate time intervals or consultation increases the risk of adverse effects. - Specific supplement-drug combinations are potentially problematic (e.g., calcium/iron with ß-blockers and levothyroxine). - Healthcare providers should be aware of patients' supplement use and inform them about potential interactions. - Labeling on dietary supplements should include interaction information	Lack of detailed test results on dietary supplement behavior in elderly populations. - Limited awareness among respondents about potential interactions between dietary supplements and drugs. - Inadequate labelling of dietary supplements regarding potential interactions. - Need for better education and communication between doctors and patients about dietary supplements
Peklar et al. (2014) [14]	Cross-sectional analysis of 8,081 community-dwelling persons aged 50 years or more in the Republic of Ireland. Data collected via in-home interviews using Computer-Aided Personal Interview software	Medication Use: 14% of respondents reported concurrent use of drugs and supplements; higher prevalence in women and those aged C75 years. - Interactions: 4.5% of concurrent users had potential major interactions; higher prevalence in older respondents. - High-Risk Groups: Women, those aged 75 years, retired individuals, those with three or more chronic conditions, and those with polypharmacy	Concurrent use of drugs and supplements is substantial and increases with age. - There is evidence of unmet needs and potential serious drug interactions. - Reliable information, public awareness, professional guidance, and practice support are needed to mitigate risks and ensure benefits. - National medicines policies should address these issues to ensure responsible supplement use and tailored pharmacovigilance	Absence of dose information for supplements - Lack of information on whether supplements were prescribed or purchased. - Limited information on reasons for taking supplements. - Absence of detailed clinical data, particularly outcomes. - Reliance on external clinical references for assessing interaction seriousness. - Need for rigorous clinical assessment in cases with multiple drugs and supplements
Sood et al. (2008) [15]	Cross-sectional, point-of-care survey of 1818 patients at Mayo Clinic (Rochester, Minn). Data collected via survey and review of patient medical records	Medication Use: 39.6% of surveyed patients reported using dietary supplements. - Interactions: 107 interactions with potential clinical significance were identified. - High-Risk Groups: Women were more likely to use dietary supplements. Patients using antithrombotic medications are at higher risk due to potential interactions with dietary supplements like garlic, valerian, kava, ginkgo, and St John's wort	The clinical implications of the study are that despite a high prevalence of potential interactions between dietary supplements and prescription medications, the actual potential for harm is low. - However, caution is advised, particularly for patients on antithrombotic medications, who should avoid certain dietary supplements. -Healthcare providers should be vigilant and advise patients to prevent potential adverse effects, even though serious harm is rare	Recall bias due to the survey nature. - Incomplete or inaccurate medical record information. - Incomplete existing information on potential interactions. - Limited generalizability to the community setting
Qato et al. (2008) [4]	Cross-sectional, nationally representative survey of 3,005 U.S. adults aged 57–85 (2005–2006). Data collected via in-home interviews and medication logs	More than half of older adults use 5 or more prescription medications, over-the-counter medications, or dietary supplements. - The oldest age group (75-85 years) has the highest prevalence of prescription medication use. - Women are more likely to use prescription medications than men. - Concurrent use of prescription and nonprescription medications is common (68%). - 11 major potential drug-drug interactions were identified, with half involving nonprescription therapies. - Men are at a higher risk for major interactions compared to women. - Gender differences in medication use are noted, with women using more prescription medications and dietary supplements	Older adults are vulnerable to medication adverse effects and drug-drug interactions due to high consumption of medications and supplements. - Concurrent use of prescription and nonprescription medications poses a risk of major drug-drug interactions. - Lack of physician awareness about nonprescription medication use exacerbates risks. - Addressing these issues is crucial for improving drug safety for seniors. - Data can help improve pharmacotherapy safety and quality for older adults	Methodological differences across studies may limit cross-study comparisons. - Data does not allow for a complete examination of the appropriateness of medication regimens due to underuse or overuse in certain populations. - Use of Thomson Micromedex classifications may lead to different estimates of drug-drug interactions if other methods were used. - Methods of classification may underestimate potential risks due to limited drug safety literature. - Focusing on only major interactions among common medications and supplements underestimates the total risk for potential interactions
Błeszynska et al. (2020) [16]	Review article	Medication Use: 30-40% of people over 65 take 5 or more drugs; 12% use 10 or more different medicines. - Interactions: One in six elderly patients is at risk of significant pharmacological interactions; 1 in 5 commonly used drugs should not be recommended. - High-Risk Groups: Elderly with multimorbidity (nearly half have at least three chronic diseases); those with hypertension, osteoarthritis, ischemic heart disease, and diabetes	Pharmacological therapy in the elderly is complicated due to advanced age, multiple morbidities, and polypharmacotherapy, leading to increased risk of drug interactions and adverse effects. - Understanding drug interactions and their consequences is crucial for optimizing pharmacotherapy and improving patient safety. Serious adverse effects include death and hospitalization due to pharmacological interactions. - The economic burden is significant, with estimated costs in the United States exceeding $200 billion annually. - Most serious drug side effects are caused by incorrect prescriptions, which are often predictable and avoidable. - Standardized tools and clinical pharmacists can help minimize these issues. - Automated systems can aid in recognizing drug interactions, but there is a need for improved dissemination of electronic information to support clinicians. - A joint effort is needed to improve pharmacotherapy quality and patient safety	The imperfection of specific criteria for drugs not indicated in geriatric patients. - Exclusion of older adults from randomized clinical trials. - Lack of accurate data on the relative risks and benefits of therapeutic measures. - Heterogeneity of the elderly population affects risk-benefit ratios. - Current tools are best used as screening tools rather than definitive measures. - Limited ability of physicians to recognize potential drug interactions. - Need for improved electronic sources of information on drug interactions
Gavronski and Volmer (2014) [17]	Cross-sectional, questionnaire-based interview of 712 Estonian residents aged 15-85 (2010-2012). Data collected via face-to-face interviews at community pharmacies and GP centers	Medication Use: 50.4% of respondents reported concomitant use of Rx and OTC medicines. - Interactions: Frequent use of Rx and OTC medicines together increases the risk of drug-drug interactions. - High-Risk Groups: Chronic and elderly patients are especially vulnerable due to increased use with age and the number of chronic diseases	Frequent concomitant use of Rx and OTC medicines, especially among chronic and elderly patients. - Increased risk of drug-drug interactions affecting efficacy and safety. - NSAIDs can reduce the effectiveness of antihypertensive medicines and cause renal impairment. - Need for healthcare professionals to provide more information on interactions and side effects. - Lack of access to complete medication records by healthcare professionals	The survey did not determine specific interactions between Rx and OTC medicines. - Insufficient knowledge of lay people about OTC medicine names. - No assessment of pharmaceutical policy impact on drug interaction identification. - Unable to determine simultaneous use of different medicines. - Potential for patients to affect medication efficacy through self-administration. - Limited access to complete medical records for healthcare professionals
Alqallaf et al. (2022) [18]	Cross-sectional study, Bahrain, sample size of 500 Bahrainis over 65 years of age. Data collected via the LMQ-3 questionnaire and semi-structured interviews distributed through hospitals, health centers, clinics, and community pharmacies using a Convenience Non-Random Sampling Design	High medication-related burden was observed in the majority of patients, with over two-thirds experiencing high burden and almost a third experiencing moderate burden. - Main drivers of burden: concerns about medicine use, side effects, and interference with daily life. - High-risk groups: technical college graduates, aged ≥75 years, using ≥9 medicines, using medicines four times a day. - Anti-diabetic medications are most commonly prescribed. - High prevalence of polypharmacy: majority using 5-6 medicines, significant number using 7-8 or more. - Females and employed individuals had higher burden rates	Healthcare professionals should engage more with elderly patients to address concerns about medicine use, side effects, and interference in daily life. - Integration of tools like the LMQ-3 into healthcare systems can help assess and manage medication burden. - Targeted interventions should be developed to reduce medication burden and improve adherence and health outcomes. - Improved communication between healthcare professionals and patients is crucial for addressing medication-related fears and improving adherence. - The LMQ-3 can be beneficial in examining the burden in patients with multiple chronic diseases	Use of phone interviews for community-dwelling elderly. - Inaccuracy in recording dietary supplements. - Lack of information on the distribution of collected responses. - Need for future studies on herb-drug interactions
Fravel et al. (2023) [5]	Secondary analysis of data from the ASPREE trial, a randomized controlled trial involving 15,732 community-dwelling older adults from Australia and the United States. Data collected via a one-off questionnaire during the final annual visit ("Milestone" study visit)	Prevalence: 57.6% of adults 20 years and older in the US use dietary supplements or CAMs; 74.3% of adults 60 years and older in the US use these products. - Risks: Increased drug interactions, medication burden, nonadherence with prescription medications, and increased out-of-pocket expenses. - High-Risk Groups: Older adults, particularly those using warfarin and aspirin, are at increased risk of serious drug interactions. - Predictors of Use: US residence, female sex, higher education level, polypharmacy, and frailty. - Commonly Used Products: Multivitamins, fish oil, calcium, and vitamin D	Increased supplement use may enhance interest in health and improve medication adherence. - Risks include increased drug interactions, medication burden, nonadherence, and financial hardship. - Healthcare providers should be involved in managing these therapies due to potential adverse effects and financial strain. - Providers should engage with patients to educate them about benefits and harms and screen for adverse effects. - Active assessment of supplement/CAM use by healthcare providers is necessary for safe and effective use	Lack of racial diversity in the cohort. - The majority of the cohort is Australian, limiting conclusions about other countries. - Selection bias due to participation in a health-related trial. - Limited data on frequency, duration, and dose of supplement/CAM use. - Need for future research with more diverse populations and detailed data collection
Campos et al. (2024) [2]	Narrative review	High prevalence of polypharmacy among the elderly increases the risk of adverse reactions and interactions with food supplements. The elderly are susceptible to adverse medication effects due to physiological and pharmacological changes. - Polypharmacy is associated with adverse health outcomes like mortality, falls, and drug interactions. - Use of FSs alongside medications increases the risk of severe drug interactions, especially with warfarin and aspirin. - Healthcare providers should assess FS use in older adults to mitigate risks. - Drug-FS interactions are significant and often overlooked in treatment plans	Increased risk of adverse reactions due to drug-food supplement interactions. - Potential for misdiagnosis of adverse drug reactions. - The elderly are more susceptible to adverse medication effects due to physiological and pharmacological changes. - Polypharmacy is associated with adverse health outcomes, including mortality, falls, and increased hospital stays. - Use of FSs can exacerbate these risks, especially with certain medications like warfarin and aspirin. - Need for educational interventions and regulatory measures to mitigate these risks	Gaps in the FS regulatory framework. - Lack of robust FS legislation. - Lack of a harmonised definition of polypharmacy. - Unclear if polypharmacy includes or does not include FSs or oral nutritional supplements. - Difficulty in comparing prevalence data. - Hinders the definition of guidelines and health policies. - Lack of safety and quality of FSs. - Lack of rigour in the distinction between FSs and oral nutritional supplements. - Inadequate safety evaluation of FSs. - Lack of education of physicians about food supplements. - Lack of communication between patients and health professionals. - Lack of data on FS consumption in the European Union. - High complexity in establishing FS consumption patterns in older people
Thurman and Mooradian (1997) [19]	Review	Vitamins taken in large dosages should be considered as drugs and regulated to prevent toxicity. - Vitamin use in the elderly is largely self-prescribed and lacks a medical basis. - Elderly individuals, especially those who are homebound or institutionalized, are at high risk for micronutrient deficiencies. - High-dose vitamin therapy is useful in some medical conditions, but there is no conclusive evidence to support widespread use. - Medicines commonly used in the elderly may deplete vitamin stores or interfere with absorption	Ascorbic acid supplementation may improve vascular integrity and manage diabetes. - Vitamin E supplementation may reduce coronary artery disease and improve insulin resistance. - Vitamin D supplementation can increase bone mineral density and is recommended for frail elderly individuals. - Pyridoxine supplementation may improve cognitive function and treat movement disorders. - Folic acid supplementation should be used with caution and ideally with cyanocobalamin to avoid masking deficiency. - High-dose vitamin therapy should be approached with caution due to potential adverse effects	There is a lack of large clinical trials to confirm the benefits of ascorbic acid supplementation. - Need for further long-term prospective studies to establish the safety and efficacy of ascorbic acid therapy. - Long-term safety of vitamin E supplementation not established. - Need for future studies on long-term safety and efficacy of large dosages of vitamins. - Current RDA for vitamin D may be inadequate for minimizing bone loss in the elderly. - Lack of conclusive evidence supporting the use of large dosages of vitamins in elderly patients
Jaqua et al. (2024) [20]	Cross-sectional study, 420 participants aged 65 and older from a geriatric primary care clinic in the United States (2020-2022). Data collected via surveys and chart reviews using digital medical records	Medication Use: 15.6% of participants experienced potential drug interactions. - Interactions: Ginkgo biloba, garlic, and calcium were common contributors to interactions among supplements; ibuprofen was a common contributor among OTC medications. - High-Risk Groups: Geriatric population, particularly those prone to polypharmacy and adverse events	The study highlights a concerning 15.6% rate of potential drug interactions among older adults using CAM and OTC medications. - There is a need for awareness among healthcare practitioners about potential interactions and standardized CAM surveys to enhance patient safety. - Improved communication and awareness about CAM use are necessary to address the disparity between patient reports and physician inquiries. - Implementing standardized CAM inquiry protocols and integrating CAM information into electronic health records can enhance patient safety and reduce adverse drug interactions	Lack of a clear definition of CAM. - Limited sample size and population representation. - Potential inaccuracies in electronic medical records. - Limited cultural, racial, and ethnic representation. - Need for further research on CAM use and its correlations with economic status, cultural background, racial differences, and ethnic differences

A highly consistent finding is the widespread use of dietary supplements by older adults, frequently in the context of polypharmacy. The reviewed studies demonstrated supplement utilization rates ranging from 23% to 82.5% in specific patient cohorts [6,9]. For example, Fravel et al. (2023) reported that 74.3% of older adults in the U.S. use these products regularly [5], while Qato et al. (2008) found that supplements are consumed by almost half of the elderly population in the U.S [4]. This supplement use occurs within a context of significant polypharmacy, with multiple studies confirming that 29-40% of older adults take five or more prescription medications [4,16], leading to a high subjective medication burden [18].

The literature consistently identifies a core group of supplements and prescription medications that pose a significant risk for interactions. Supplements with anticoagulant or antiplatelet properties, such as garlic, ginkgo biloba, fish oil, and antithrombotic medications like warfarin and aspirin, were the most frequently cited interactions [6,11,20]. Minerals like calcium and iron, which can chelate and reduce the absorption of essential medications like levothyroxine and beta-blockers, comprise another involved category [13]. Consequently, several high-risk subgroups were consistently identified, including adults aged ≥75 years, women, patients with multiple chronic conditions (especially cardiovascular disease), and those with established polypharmacy [5,10,14].

Finally, a well-documented communication gap between patients and healthcare providers significantly increases the risk of adverse events. Canter (2004) found that older adults frequently fail to report their supplement use to doctors [12]. Studies have quantified this gap, revealing that clinicians often fail to proactively ask about supplement use, even when patients disclose other complementary and alternative medicine (CAM) practices. This oversight results in incomplete medication histories and missed opportunities to prevent harm [20].

This review synthesizes a compelling, albeit complex, body of evidence from 16 international studies conducted over nearly three decades. The findings converge on three critical and interrelated themes: the high prevalence of concurrent supplement and medication use in older adults, the identification of specific pharmacological "hotspots" for interactions, and the systemic breakdown in patient-provider communication that turns potential risks into actual clinical hazards.

The analysis reveals a critical convergence of risk factors: high rates of dietary supplement use (23-82.5%) coinciding with prevalent polypharmacy, creating optimal conditions for adverse events [4,5,9]. While studies like those by Qato et al. and Błeszyńska et al. (2020) quantify polypharmacy [4,16], Alqallaf et al. (2022) add a crucial dimension by highlighting the high subjective burden this creates for patients, driven by fears of side effects and interference with daily life [18]. This context suggests that patients are not just passive recipients of complex regimens but are actively seeking alternatives, like supplements, which they may perceive as safer.

Our analysis identifies two particularly high-risk pharmacological interactions: 1. Pharmacodynamic interactions: Multiple studies consistently demonstrate clinically significant effects when hemostasis-altering supplements (garlic, ginkgo biloba, fish oil) are combined with antithrombotic agents (warfarin, aspirin) [6,11,20]. This represents the most robustly documented interaction category. 2. Pharmacokinetic interactions: Mineral-containing supplements (calcium, iron) frequently chelate essential medications like levothyroxine, significantly compromising drug absorption and therapeutic efficacy [12].

However, a nuanced point of discussion emerges when contrasting potential versus actual harm. While studies by Chan et al. (2012) [10] and Jaqua et al. (2024) [20] report that 12-16% of older adults face potential interactions, Sood et al. (2008) [15] provide a crucial counterpoint, suggesting that the actual incidence of serious clinical harm may be lower. This distinction is vital: while the potential for interactions is vast, the real-world clinical consequences may be concentrated within specific, high-risk combinations. These findings necessitate a paradigm shift from generic cautionary statements to focused clinical surveillance of high-risk supplement-drug combinations.

A clinically significant revelation from our review is that communication failures between patients and providers represent the critical pathway through which theoretical interaction risks manifest as preventable harm. The evidence demonstrates this is not simply a matter of patient nondisclosure - multiple studies [12,20] document clinicians' systematic failure to proactively screen for supplement use, despite established risks. This communication void renders even the most diligent clinician unable to accurately assess a patient's true risk profile, making preventable interactions almost inevitable.

Clinical implications

The accumulated evidence compellingly demonstrates that preventing supplement-drug interactions in older adults requires fundamental practice transformation, from passive awareness to systematic prevention. The cornerstone of this shift must be the implementation of routine and standardized screening for all supplement use. This necessitates more than just a passing question; it requires integrating a dedicated supplement history into the standard workflow of every clinical encounter, particularly during high-risk moments like medication reconciliation. To facilitate this, healthcare systems should adopt the recommendation of Jaqua et al. (2024) to implement standardized screening tools directly within the electronic health record [20]. This systemic approach ensures that a complete medication profile, inclusive of all supplements, is consistently available to the entire care team.

Building on this foundation of enhanced screening, targeted patient education, and interprofessional collaboration are essential. Partial warnings about interactions are insufficient; education must be targeted, focusing on the common, high-risk combinations identified in this review, such as the increased bleeding risk with warfarin and ginkgo, or the impaired absorption of levothyroxine with calcium supplements [13]. Furthermore, the sheer complexity and prevalence of polypharmacy and drug-related problems (DRPs), as documented by Chan et al. (2012) [10], underscore that managing these risks cannot be the sole responsibility of a single clinician. The evidence robustly supports scaling pharmacist-led medication therapy management services to outpatient and community settings. As medication experts, pharmacists are uniquely positioned to conduct comprehensive medication reviews, detect clinically significant pharmacokinetic and pharmacodynamic interactions, and advise both patients and prescribers on risk mitigation strategies. This expansion would establish an essential safeguard for older adults navigating complex medication regimens.

Strengths and limitations

The body of evidence synthesized in this review possesses several notable strengths. The geographic and temporal breadth of the included studies, spanning nearly three decades and multiple continents, demonstrates that the challenge of supplement-drug interactions in older adults is a persistent and global phenomenon. The inclusion of several large-scale, population-based surveys provides robust data on the high prevalence of concurrent use [4,5]. Furthermore, the consistency of findings across diverse studies repeatedly identifying agents like warfarin and supplements like garlic as high-risk strengthens the validity of these specific warnings.

These strengths are counterbalanced, however, by significant limitations within the primary literature. The most critical weakness is the predominance of cross-sectional study designs, which can establish association but not causation between supplement use and adverse clinical events. Methodological heterogeneity is also a major challenge; studies define and assess supplement use and drug interactions differently, precluding a quantitative meta-analysis and complicating direct comparisons. Heavy reliance on self-reported data introduces recall and social desirability biases. Finally, geographical gaps are also noted due to limited studies from low- and middle-income countries, where most older adults globally reside.

This literature review was designed with methodological rigor, employing a structured search strategy and a systematic screening process to minimize selection bias. Including both original research and review articles, it provides a holistic synthesis of primary data and expert interpretation. Nonetheless, the review has its own inherent limitations. The decision to conduct a narrative synthesis, necessitated by the heterogeneity of the included studies, is more susceptible to interpretive bias than a quantitative meta-analysis. Potential introduction of language bias is also a concern, as the search was restricted to English-language publications. Lastly, the possibility of publication bias, wherein studies that find no significant interactions are less likely to be published, cannot be dismissed and should be considered when interpreting the overall risk profile presented herein.

Future research directions

This review identifies critical knowledge gaps that chart essential directions for future investigation to enhance medication safety in older adults. Foremost is the need to move beyond the limitations of associative cross-sectional data, prioritizing prospective, longitudinal cohort studies capable of tracking patients over time to establish relevant causal links between specific supplement-drug combinations and clinical outcomes. This approach will allow for the quantification of the absolute risk of adverse events, moving beyond the identification of potential interactions to understanding their real-world clinical impact. Concurrently, these observational studies should be complemented by mechanistic pharmacokinetic and pharmacodynamic research to better understand and predict the interactions of less-studied but commonly used supplements.

Alongside foundational research, there is an urgent need for intervention-based studies designed to test practical solutions. The critical role of the communication gap highlights a need to rigorously evaluate the effectiveness of strategies such as pharmacist-led medication therapy management programs, the integration of automated interaction alerts into electronic health records, and targeted patient education campaigns. Furthermore, future research must address the stark lack of data from low- and middle-income countries, where healthcare systems and cultural beliefs about supplements may differ significantly. Studies should also focus on particularly vulnerable settings, such as during transitions of care between hospital and community, where the risk of medication errors is highest.

Finally, to enable more powerful and precise evidence synthesis in the future, the field would benefit from a concerted effort to develop and adopt standardized methodologies. This includes creating consensus-based common data elements for defining and classifying supplement use and establishing a tiered system for rating the clinical severity of potential drug interactions. Such standardization would pave the way for future quantitative meta-analyses, which can provide more robust risk estimates and strengthen the evidence-based guidelines needed to ensure medication safety for a growing global population of older adults. Additionally, as the demographic imperative of an aging population becomes increasingly urgent, heeding the call for a deeply integrated, competencies-driven curriculum is paramount. Safeguarding the standard of care for older patients globally requires the seamless integration of geriatric medicine topics throughout undergraduate medical training [21].

## Conclusions

This review highlights a significant and persistent public health challenge: the underrecognized risks of supplement-drug interactions in older adults. Given the rapidly growing geriatric population, the combination of high supplement use, polypharmacy, and poor clinician-patient communication leads to preventable hazards, particularly involving anticoagulants and thyroid medications. While potential interactions are numerous, clinically significant adverse effects cluster within identifiable high-risk combinations. Clinicians should implement proactive measures, including routine supplement screening as part of standard history-taking, targeted patient counseling, and interprofessional collaboration, to reduce risks. However, the current evidence is limited given the cross-sectional study designs and methodological variability, emphasizing the need for future longitudinal and intervention-based research. Developing standardized research methodologies and ensuring global representation are prerequisites for creating evidence-based clinical guidelines to optimize medication safety in aging populations.
